# Risikowahrnehmung und Informationsverhalten von Meinungsführenden im Lebensmittelbereich

**DOI:** 10.1007/s00103-020-03252-2

**Published:** 2020-12-08

**Authors:** Ann-Kathrin Lindemann, Katrin Jungnickel, Gaby-Fleur Böl

**Affiliations:** Abteilung Risikokommunikation, Bundesinstitut für Risikobewertung, Max-Dohrn-Str. 8–10, 10589 Berlin, Deutschland

**Keywords:** Risikowahrnehmung, Meinungsführerschaft, Risikokommunikation, Informationsverhalten, Lebensmittelsicherheit, Risk perception, Opinion leadership, Risk communication, Information behaviour, Food safety

## Abstract

Meinungsführer*innen sind Personen, die in ihrem sozialen Umfeld die Meinungen, Einstellungen oder das Verhalten von anderen Personen beeinflussen können. Sie können insbesondere in sozialen Netzwerken im Internet auch größere Zielgruppen erreichen und stellen deshalb eine zentrale Zielgruppe für die Risikokommunikation dar. Eine wichtige Voraussetzung für eine gelungene Risikokommunikation ist eine genaue Kenntnis der Risikowahrnehmung der entsprechenden Zielgruppe, um diese bei der Entwicklung von Kommunikationsmaßnahmen berücksichtigen zu können. In der vorliegenden Studie wurde deshalb untersucht, inwieweit es einen Zusammenhang zwischen der Risikowahrnehmung von Lebensmittelthemen und themenspezifischer Meinungsführerschaft gibt. Hierzu wurde eine repräsentative Telefonbefragung der Bevölkerung in Deutschland durchgeführt (*n* = 1001). Demnach weisen Meinungsführer*innen bei einigen Themen eine etwas höhere Risikowahrnehmung gegenüber Lebensmittelrisiken auf als Nicht-Meinungsführer. Sie sind zudem an diesen Themen deutlich stärker interessiert und greifen häufiger über bestimmte Medien auf Informationen zu Lebensmittelthemen zu. Meinungsführer*innen sind zudem häufiger in sozialen Medien aktiv und können so im dortigen Stimmengewirr durch ihr spezielles Wissen und ihre Einschätzungen eine wertvolle Orientierung für ihr soziales und digitales Umfeld bieten.

## Abstract (en)

Opinion leaders are people who can influence the opinions, attitudes or behaviours of others in their social environment. Especially in social networks on the Internet, they can also reach larger audiences and therefore represent a crucial target group for risk communication. An important prerequisite for successful risk communication is a precise knowledge of the risk perception of the relevant target group, so that this can be taken into account when developing communication measures. The present study therefore investigated the extent to which there is a connection between the risk perception of food issues and issue-specific opinion leadership. For this purpose, a representative telephone survey of the population in Germany was conducted (*n* = 1001). According to this, opinion leaders have a slightly higher risk perception of food risks for some topics than non-opinion leaders. They are also much more interested in these topics and access information on food issues more frequently via specific media. Opinion leaders also tend to be more active in social media. In the buzz of social media, they can thus offer valuable orientation for their social and digital environment through their special knowledge and opinions.

## Meinungsführerschaft im Kontext des Medienwandels

Eine gute Ernährung mit sicheren Lebensmitteln ist für Verbraucher*innen in Deutschland ein zentrales Gesundheitsthema. Wie eine repräsentative Umfrage des Bundesinstituts für Risikobewertung zeigt, stellt für 14 % der Befragten eine ungesunde oder falsche Ernährung eines der drei größten gesundheitlichen Risiken für Verbraucher*innen dar – lediglich Klima- und Umweltbelastungen wurden auf die offen gestellte Frage noch häufiger genannt [1, S. 5]. Ungesunde oder belastete Lebensmittel sowie Schad- und Zusatzstoffe zählen für jeweils 8 % der Befragten zu den größten Risiken. Diese Ergebnisse verdeutlichen, wie wichtig eine wirksame Risikokommunikation im Bereich der Lebensmittelsicherheit ist. Um zielgruppengerechte Kommunikationsmaßnahmen entwickeln zu können, ist jedoch zunächst eine genaue Kenntnis der Risikowahrnehmung notwendig, die von verschiedenen Faktoren wie spezifischen Eigenschaften des Risikos oder der entsprechenden Zielgruppe abhängt [2]. Für die Risikokommunikation können dabei Meinungsführer*innen eine besondere Position einnehmen. Als Meinungsführer*innen werden generell Personen bezeichnet, die in ihrem sozialen Umfeld die Meinungen, Einstellungen oder das Verhalten von anderen Personen beeinflussen können [3, 4]. Durch das ihnen entgegengebrachte Vertrauen können sie andere über Risiken aufklären und so auf die Risikowahrnehmung ihres Umfeldes einwirken.

Die Forschung zur Meinungsführerschaft blickt auf eine lange Tradition zurück [3–5]. Meinungsführer*innen wurden erstmals im Jahr 1944 in der People’s-Choice-Studie [6, 7] identifiziert, in der deutlich wurde, dass Freund*innen und Bekannte einen relativ großen Einfluss auf die Wahlentscheidung der Studienteilnehmenden hatten, während der Einfluss der Medienberichterstattung vergleichsweise gering ausfiel. Besonders politisch wenig interessierte Personen ließen sich stark von ihrem sozialen Umfeld beeinflussen. Hieraus leiteten die Autoren einen sogenannten „Zweistufenfluss“ der Kommunikation ab, wonach Medieninformationen zunächst von politisch besonders interessierten Personen rezipiert werden, bevor diese sie wiederum an die weniger interessierten Personen weitertragen. Die politisch besonders interessierten oder aktiven Personen waren dabei keine Wahlkampfmitarbeiter*innen, Journalist*innen oder Vertreter*innen von Organisationen oder Firmen, sondern Privatpersonen aus allen sozialen Schichten mit einem heterogenen soziodemografischen Hintergrund [7].

Die Idee des Zweistufenflusses der Kommunikation wurde von Menzel und Katz (1955; [8]) zu einem Mehrstufenfluss weiterentwickelt, um auch den Interaktionen zwischen Meinungsführer*innen Rechnung zu tragen. In den Arbeiten zur Diffusionstheorie von Rogers (2003; [9]) wird zudem der Einfluss von Meinungsführer*innen bei der Verbreitung und Adaption von Innovationen in Gesellschaften deutlich. Aufgrund dieser verschiedenen Anknüpfungspunkte an das Konzept der Meinungsführerschaft ist es nicht überraschend, dass Meinungsführerschaft nicht nur in den Sozialwissenschaften, sondern auch in Wirtschaftswissenschaft [10, 11], Medizin [12, 13] und – nicht zuletzt durch das Aufkommen der sozialen Medien – auch vermehrt in der Informatik [14, 15] untersucht wird.

Meinungsführerschaft ist dabei nicht zwingend eine Eigenschaft, die sich über alle Lebensbereiche einer Person erstreckt, sondern auch nur einzelne Themengebiete wie Gesundheit umfassen kann. Bei dieser themenspezifischen Meinungsführerschaft ist der Einfluss der Person vor allem auf ihr besonderes Interesse und Wissen bezüglich dieses Themengebiets zurückzuführen. Eine allgemeine Meinungsführerschaft, die über einzelne Themenbereiche hinausgeht, stützt sich dagegen eher auf themenunspezifische Aspekte wie das Charisma einer Person [16]. Obwohl insbesondere die themenspezifische Meinungsführerschaft hinsichtlich Gesundheit und Ernährung bereits an unterschiedlichen Aspekten und Fragestellungen untersucht wurde [17–19], gibt es bislang keine Studie, in der Meinungsführer*innen im Gesundheitsbereich hinsichtlich ihrer Risikowahrnehmung charakterisiert wurden. Dies könnte jedoch für die Risikokommunikation hilfreich sein.

Durch die steigende Bedeutung des Internets und der sozialen Medien – sowohl als Kommunikationskanal als auch als Nachrichtenquelle [20–22] – können Meinungsführer*innen nicht nur einzelne Freund*innen oder Bekannte über Gespräche informieren, sondern haben das Potenzial, über solche Plattformen auch größere Reichweiten zu erzielen. Dabei können sie auf unterschiedliche Art und Weise Einfluss ausüben. Zum einen können Meinungsführer*innen eigene Einschätzungen posten und in der direkten Interaktion mit anderen Nutzer*innen ihr Wissen und ihre Einschätzungen weitergeben [23]. Darüber hinaus können sie auch als Gatekeeper (Torwächter*innen) fungieren, indem sie bewusst auswählen, welche Inhalte sie an ihr soziales Netzwerk aus Followern oder Freund*innen weiterleiten und welche nicht. Ohne sich dabei selbst explizit äußern zu müssen, können Meinungsführer*innen so bereits durch das Teilen und Weiterleiten von Informationen aus Medien oder anderen Quellen die Wahrnehmung und Einstellungen ihrer Follower beeinflussen. Da Onlinecommunitys und soziale Medien bei der Suche nach Informationen im Gesundheitsbereich gerade für junge Menschen eine zentrale Rolle spielen [24, 25], ist es denkbar, dass Meinungsführer*innen hier als Multiplikatoren wirken und Informationen zu Gesundheitsthemen und deren Risikoeinschätzung an ihr soziales Umfeld weitergeben. Ein besonderes Augenmerk soll in dieser Studie deshalb auch auf die Nutzung von sozialen Medien durch Meinungsführer*innen und Nicht-Meinungsführer*innen gelegt werden. Dabei muss jedoch berücksichtigt werden, dass diese Informationsweitergabe nicht unbedingt neutral verläuft, sondern von den eigenen Wahrnehmungen und Einstellungen der Meinungsführer*innen beeinflusst werden kann. Dies kann dazu führen, dass Meinungsführer*innen auch die Risikowahrnehmung ihrer Mitmenschen beeinflussen.

## Zusammenhang von Risikowahrnehmung und Meinungsführerschaft

In der Forschung existieren unterschiedliche Ansätze, um die individuelle Risikowahrnehmung zu erklären [26]. Bereits seit den späten 1970er-Jahren begann die Forschungsgruppe um Fischhoff, Slovic und Lichtenstein [27] mit der Entwicklung des „Risk Perception Models“, welches basierend auf dem psychologischen Paradigma zu erklären versucht, welche spezifischen Eigenschaften von Risiken die Risikowahrnehmung beeinflussen können. Demnach führen wissenschaftliche Unsicherheit, eine breite Medienberichterstattung sowie eine zeitliche Verzögerung der Auswirkungen eines Risikos zu einer hohen Risikowahrnehmung, während bekannte Risiken, ein Gefühl von Kontrolle über die Situation sowie freiwillig eingegangene Risiken mit einer geringeren Risikowahrnehmung verknüpft sind [2, 28]. Medienberichterstattung wird in diesem Modell vor allem als Verstärker der Risikowahrnehmung gesehen, während nach dem „Social Amplification of Risk Framework“ (soziale Verstärkung von Risiken) von Kasperson und Kollegen (1988; [29]) auch eine abschwächende Wirkung der Medienberichterstattung auf die Risikowahrnehmung möglich ist. Nach diesem Ansatz durchlaufen Risikoinformationen oft mehrere Stationen, bis sie vom Sender beim Empfänger angekommen sind. Welche konkreten Stationen eine Information durchläuft und wie die Information dabei von den Stationen verändert werden, kann demnach beeinflussen, ob die Risikowahrnehmung erhöht oder abgeschwächt wird [29].

Obwohl der Social Amplification of Risk Framework lange vor der Entstehung von sozialen Netzwerken im Internet entwickelt wurde, lassen sich seine Grundprinzipien auch auf soziale Medien übertragen [30]. So kann bereits die Verwendung von anderen Formulierungen in den Kommentaren zu den zitierten Originalbeiträgen in sozialen Netzwerken dazu führen, dass das Risiko verzerrt dargestellt wird [30]. Häufig werden jedoch auch Risikodarstellungen aus den traditionellen Medien übernommen und in sozialen Netzwerken weiterverbreitet [31]. Dabei kann sich das Ausmaß der Verstärkung bzw. Abschwächung von Risikoeinschätzungen unter anderem je nach Region, Sprache und Plattform unterscheiden [32].

Dies macht deutlich, dass auch die Art der Vermittlung von Risikoinformationen einen Einfluss auf die Risikowahrnehmung hat. So erhalten beispielweise negative Informationen häufig eine deutlich größere Aufmerksamkeit als positive Botschaften und werden zudem auch meist besser erinnert [33]. Nach diesem „Negative Dominance Model“ kann so selbst eine vermeintlich neutrale Berichterstattung über Risiken zu einer höheren Risikowahrnehmung führen [26]. Neben der Botschaft kann jedoch auch die Quelle ausschlaggebend für die Wirkung von Risikoinformationen sein, wobei hier vor allem das wahrgenommene Vertrauen in den jeweiligen Kommunikator entscheidend ist („Trust Determination Model“; [26]). Während sich die Forschung im Bereich Vertrauen und Risikowahrnehmung häufig auf das Vertrauen in Institutionen und staatliche Regulierungen konzentriert [34–36], können generelle Prinzipien hiervon auch auf das Vertrauen in Einzelpersonen – wie Meinungsführer*innen – übertragen werden. So kann Vertrauen in der Regel nur über einen längeren Zeitraum aufgebaut werden, wobei nicht nur die Validität der weitergegebenen Informationen entscheidend ist, sondern auch die Art und Weise, mit der diese Informationen kommuniziert werden [37]. Das Vertrauen kann jedoch wiederum geschmälert werden, wenn beispielsweise der Eindruck entsteht, dass der Kommunikator eigene Interessen verfolgt [38]. Da Meinungsführer*innen in der Regel keine kommerziellen Interessen verfolgen, genießen sie häufig mehr Vertrauen. Daher können sie nicht nur durch die Weitergabe von Informationen die Risikowahrnehmung anderer Personen beeinflussen, sondern auch durch die Kommunikation ihrer eigenen Einschätzungen sowie durch ihr (Kommunikations‑)Handeln in- und außerhalb dieses Risikothemas.

Vertrauen spielt auch eine entscheidende Rolle bei der Risikowahrnehmung im Bereich der Lebensmittelsicherheit. So zeigt die Metaanalyse von Nardi und Kolleg*innen [39], dass unter anderem Vertrauen in die Regierung und Vertrauen in die Akteure der Lieferketten zu einer niedrigeren Risikowahrnehmung im Lebensmittelbereich führt. Auch das subjektive Gefühl, mehr über Themen im Lebensmittelbereich zu wissen, ist tendenziell mit einer niedrigeren Risikowahrnehmung verknüpft. Schließlich können auch individuelle Faktoren wie die eigene Einstellung oder soziodemografische Faktoren die Risikowahrnehmung im Lebensmittelbereich beeinflussen [39].

Der Zusammenhang zwischen Risikowahrnehmung und Meinungsführerschaft im Lebensmittelbereich wurde jedoch bislang noch nicht näher untersucht. Somit ist bisher nicht bekannt, inwiefern sich die Risikowahrnehmung von Meinungsführer*innen sowie ihr Informationsverhalten in Bezug auf Risikoinformationen von Nicht-Meinungsführer*innen unterscheiden. Zudem wurde auch noch nicht näher untersucht, wie Meinungsführer*innen im Lebensmittelbereich soziale Medien für die Weitergabe von Informationen nutzen.

Um hierauf Antworten zu finden, wurden mithilfe einer repräsentativen Telefonbefragung Meinungsführer*innen im Bereich Lebensmittelsicherheit identifiziert und charakterisiert. Die hier präsentierten Ergebnisse sind dabei Teil einer größeren Studie zum Zusammenhang zwischen allgemeiner und themenspezifischer Meinungsführerschaft und Risikowahrnehmung [40].

## Methodik

Im Rahmen dieser Studie wurde durch das Meinungsforschungsinstitut Kantar EMNID eine repräsentative Telefonbefragung durchgeführt. Der Erhebungszeitraum erstreckte sich vom 05.12.2018 bis zum 05.01.2019. Die Grundgesamtheit wurde aus allen deutschsprachigen Personen über 14 Jahren gebildet, die entweder über mindestens einen Mobil- oder einen Festnetzanschluss in Privathaushalten in Deutschland erreichbar waren. Bei der Erstellung der Telefonstichprobe wurde der Dual-Frame-Ansatz berücksichtigt, sodass die Stichprobe sowohl Festnetzanschlüsse als auch Mobilfunknummern enthielt. Durch eine zufällige Generierung der Telefonnummern über Random Digit Dialing wurde sichergestellt, dass auch nicht eingetragene Anschlüsse erreicht werden konnten. Da durch eine Mobilfunknummer in der Regel nur eine Person erreicht werden kann, erfolgten hier keine weiteren Schritte für die Stichprobenziehung. Beim Anruf einer Festnetznummer wurde hingegen zunächst nach der Haushaltsgröße gefragt und – sofern der Haushalt mehr als eine Person umfasste – mittels des Schwedenschlüssels [41] die bzw. der Befragungsteilnehmer*in ausgewählt. Insgesamt nahmen 1001 Personen an der Befragung teil, wobei 801 Personen davon über Festnetzanschlüsse erreicht wurden. Um trotz Stichprobenausfällen die Repräsentativität der Befragung zu gewährleisten, wurden die Daten anhand des zu diesem Zeitpunkt aktuellen Mikrozensus des Statistischen Bundesamtes (2018) nach Bundesland, Ortsgröße, Geschlecht, Alter, Schulbildung und Haushaltsgröße gewichtet (Redressement).

**Figure 1:**
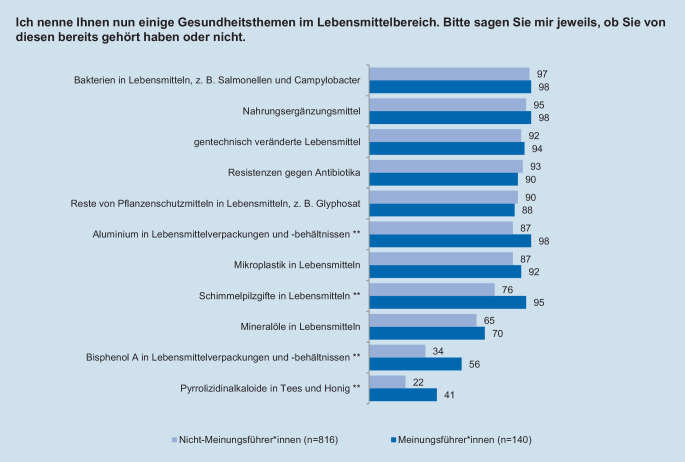

**Figure 2:**
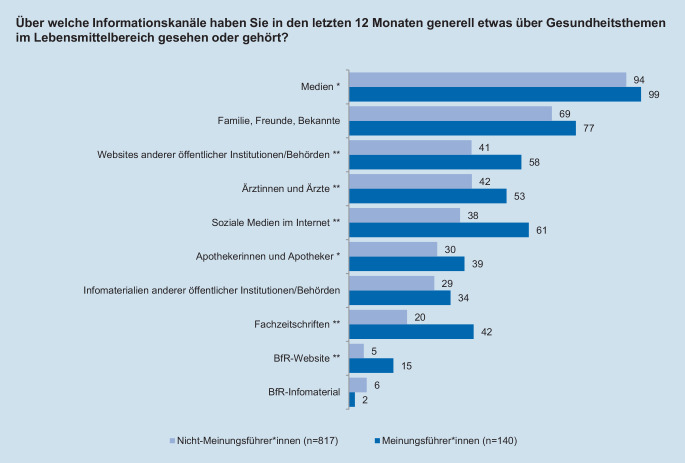

**Figure 3:**
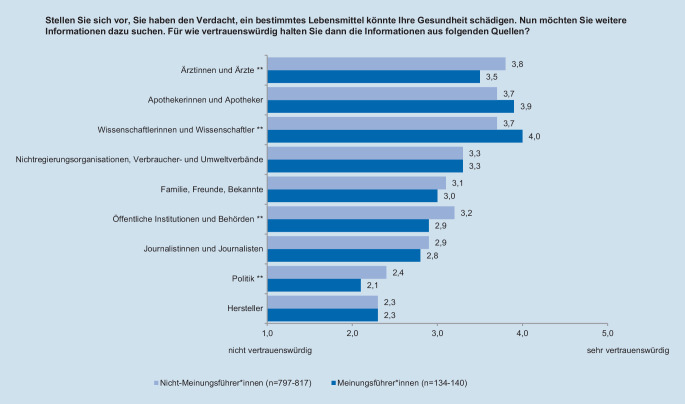

Während der Interviews beantworteten die Teilnehmenden unter anderem Fragen zu ihrer Risikowahrnehmung im Bereich Lebensmittelsicherheit, ihrer allgemeinen Risikobereitschaft sowie zu ihrem Interesse für Gesundheitsthemen im Lebensmittelbereich. Die Risikowahrnehmung wurde über 2 Fragen erhoben. Zunächst sollten die Befragten für verschiedene Risikothemen angeben, ob sie bereits vor der Teilnahme an der Studie von diesen gehört hatten (Abb. 1). Danach sollten die Teilnehmenden bei jedem ihnen bekannten Risikothema auf einer Skala von 1 bis 5 angeben, inwieweit sie darüber beunruhigt sind. Auch das Themeninteresse wurde 5‑stufig erhoben. Dabei sollten die Befragten einschätzen, wie stark sie sich für Gesundheitsthemen im Lebensmittelbereich interessieren. Zur Messung der allgemeinen, themenunabhängigen Risikobereitschaft der Befragten wurde – in Anlehnung an die Fragebögen des sozioökonomischen Panels vom Deutschen Institut für Wirtschaftsforschung [42] – eine Frage mit einer 11-stufigen Skala verwendet, auf der sich die Personen selbst einschätzen konnten (Tab. 3). Da die durch eine Selbstauskunft gemessene Risikobereitschaft der Befragten auch langfristig relativ stabil ist, wurde diese Messmethode anderen, weniger reliablen Verfahren wie Lotteriesystemen vorgezogen [43, 44]. Um die Anzahl und Art der genutzten Informationsquellen zu Gesundheitsthemen im Lebensmittelbereich zu erfassen, wurde für 10 Informationsquellen erfasst, ob die Befragten dort in den letzten 12 Monaten etwas über Gesundheitsthemen im Lebensmittelbereich gesehen oder gehört haben (Abb. 2). Außerdem wurde das Vertrauen in verschiedene Institutionen als Informationsquellen ebenfalls über eine 5‑stufige Skala erhoben (Abb. 3).

Um Meinungsführer*innen zu identifizieren, wurden in der bisherigen Forschung unterschiedliche Methoden verwendet, wobei 3 Methoden dabei dominieren. In Onlinestudien sowie Arbeiten zu sozialen Medien aus dem Bereich Informatik werden Meinungsführer*innen häufig durch spezifische *Kennzahlen oder Algorithmen* identifiziert, während in sozial- und wirtschaftswissenschaftlichen Studien die *Selbsteinschätzung* der Proband*innen dominiert. Bei der Selbsteinschätzung füllen die Studienteilnehmenden Fragebögen über ihre eigene Person aus, in denen bekannte Eigenschaften von Meinungsführer*innen über Skalen erfasst werden. Im Bereich Medizin und Gesundheit dominiert dagegen die *Fremdeinschätzung* von Meinungsführer*innen, bei der Personen zu ihrem sozialen Umfeld befragt und aus diesen Angaben die relevanten Meinungsführer*innen identifiziert werden [3]. Um den Zusammenhang von Meinungsführerschaft und anderen individuellen Merkmalen wie der Risikowahrnehmung und dem Informationsverhalten zu analysieren, wurden Meinungsführer*innen in dieser Studie mithilfe der Selbsteinschätzung identifiziert. Die themenspezifische Meinungsführerschaft zu Gesundheitsthemen im Lebensmittelbereich wurde mit einer Skala erhoben, in der die im systematischen Review von Jungnickel [3, 4] identifizierten Indikatoren für Meinungsführerschaft mit jeweils einem Item abgefragt wurden. Die Befragten sollten dabei auf einer Skala von 1 bis 5 angeben, inwieweit sie der Aussage zustimmen, wobei höhere Werte einer höheren Zustimmung zu dem jeweiligen Item entsprachen (Tab. 1). Auf der Skala für die themenspezifische Meinungsführerschaft wurde eine Reliabilität von 0,799 (Cronbach’s Alpha) erreicht. Die durchschnittliche themenspezifische Meinungsführerschaft lag bei einem Wert von M = 2,8 (SD = 0,9).

**Table 1:** 

Kriterium	Item	*n*	M	SD
Interpersonale Kommunikation	Ich spreche mit Freunden oder Bekannten über Gesundheitsthemen im Lebensmittelbereich	997	3,0	1,3
Online-Aktivität^a^	Ich schreibe online in sozialen Medien im Internet Beiträge oder Kommentare zu Gesundheitsthemen im Lebensmittelbereich	977	1,2	0,7
Informationssuche (Wissen/Expertise)	Ich informiere mich über aktuelle Erkenntnisse und Empfehlungen zum Thema Gesundheit im Lebensmittelbereich	989	3,0	1,2
Informationen geben	Wenn ich etwas Neues über Gesundheitsthemen im Lebensmittelbereich erfahre, erzähle ich meinen Freunden oder Bekannten davon	995	3,3	1,3
Rat geben	Wenn es um Gesundheitsthemen im Lebensmittelbereich geht, fragen mich meine Freunde um Rat	994	2,4	1,3
Einfluss auf Meinung	Wenn ich mir über ein bestimmtes Gesundheitsthema im Lebensmittelbereich eine Meinung gebildet habe, kann ich auch meine Freunde und Bekannten davon überzeugen	989	3,0	1,1
Einfluss auf Verhalten	Durch meine Ratschläge haben Freunde und Bekannte ihr Kauf‑, Koch- oder Ernährungsverhalten geändert	977	2,3	1,2

Hinweis: Abweichungen hinsichtlich der Fallzahl entstehen durch das Antwortverhalten der Befragten

*1* = stimme gar nicht zu, *5* = stimme voll und ganz zu

*n* Fallzahl; *M* Mittelwert; *SD* Standardabweichung

^a^aufgrund geringer Varianz aus der Skala ausgeschlossen

Als Meinungsführer*innen wurden in dieser Studie alle Befragten definiert, die auf der Meinungsführerskala mindestens eine Standardabweichung über dem Mittelwert liegen – dies ist ein häufig genutztes Verfahren zur Segmentierung dieser Gruppe [3]. Somit wurden 14 % der Befragten als themenspezifische Meinungsführer*innen eingestuft. Demografisch unterscheiden sich Meinungsführer*innen und Nicht-Meinungsführer*innen nicht voneinander. So konnten weder bezüglich der Altersstruktur, Geschlechterverteilung oder des Bildungsniveaus signifikante Unterschiede zwischen den beiden Gruppen festgestellt werden.

Aus der Frage zur aktiven Nutzung von sozialen Medien sowie den Angaben der Befragten zum Informationsverhalten wurde eine neue Variable erstellt. Dazu wurden alle Personen, die bei der Aussage: „Ich schreibe online in sozialen Medien im Internet Beiträge oder Kommentare zu Gesundheitsthemen im Lebensmittelbereich“, einen anderen Wert als „stimme gar nicht zu“ angegeben hatten, als zumindest gelegentlich „Soziale-Medien-Aktive“ eingruppiert (13 %). Personen, die zwar nicht in diese Gruppe fielen, dafür jedoch angaben, dass sie soziale Medien als Informationsquelle für Gesundheitsthemen nutzen, wurden als „Soziale-Medien-Passive“ eingestuft (29 %). Insgesamt 57 % der Befragten schrieben weder aktiv Beiträge und Kommentare in sozialen Medien noch nutzten sie diese als Informationsquelle für Gesundheitsthemen.

## Ergebnisse

In den Ergebnissen lassen sich zunächst Hinweise auf einen Mehrstufenfluss der Kommunikation zu Gesundheitsthemen im Lebensmittelbereich finden. 74 % der Befragten stimmten der Aussage, dass sie neue Informationen zum Thema Gesundheit auch an Freund*innen und Bekannte weitergeben, zumindest tendenziell zu. 45 % der Befragungsteilnehmer*innen waren zudem der Auffassung, dass ihre Freund*innen und Bekannten ihrem Rat zu einem veränderten Ernährungsverhalten gefolgt sind. Gleichzeitig werden private Kontakte von über zwei Dritteln der Befragten als Quelle für Gesundheitsinformationen genannt, lediglich Medien wurden noch häufiger als Informationsquelle genutzt. Freund*innen und Bekannte, und unter ihnen besonders die Meinungsführer*innen, können somit in Bezug auf Lebensmittel in ihrem privaten Umfeld eine zentrale Rolle als Informationsquelle einnehmen.

Um mehr über Meinungsführer*innen zu erfahren, wurde zunächst untersucht, inwieweit sich Meinungsführer*innen und Nicht-Meinungsführer*innen hinsichtlich ihrer Risikowahrnehmung von ausgewählten Gesundheitsthemen im Lebensmittelbereich unterscheiden. Hier zeigten sich bereits erste Unterschiede in der Bekanntheit der verschiedenen Risikothemen. So kannten die als Meinungsführer*innen klassifizierten Personen im Durchschnitt etwas mehr Risikothemen (MW = 9,2; SD = 1,8) als andere Personen (MW = 8,4; SD = 1,6). Wie in Abb. 1 deutlich wird, wiesen Themen wie Bakterien in Lebensmitteln oder Nahrungsergänzungsmittel generell eine hohe Bekanntheit unter den Befragten auf. Deutliche Unterschiede zeigten sich bei den weniger bekannten Themen wie Schimmelpilzgiften in Lebensmitteln oder Pyrrolizidinalkaloiden in Tees und Honig. Bei diesen Themen lag der Anteil der Personen, die von dem Thema bereits gehört hatten, bei den Meinungsführer*innen signifikant höher als bei den anderen Personen.

Doch nicht nur hinsichtlich der Bekanntheit der Risikothemen zeigten sich Unterschiede. So wurde in der Analyse auch deutlich, dass Meinungsführer*innen über 7 von 13 Risikothemen signifikant beunruhigter sind als andere Personen. Anders als bei der Bekanntheit trat diese Differenz jedoch nicht nur bei bislang weniger bekannten Themen auf. Wie in Tab. 2 dargestellt, zeigte sich der stärkste Effekt bei der Beunruhigung durch Rückstände von Pflanzenschutzmitteln wie Glyphosat. Aber auch bei Themen wie Schimmelpilzgiften in Lebensmitteln und gentechnisch veränderten Lebensmitteln waren Meinungsführer*innen etwas besorgter als Nicht-Meinungsführer*innen. Risiken durch Nahrungsergänzungsmittel oder Küchenhygiene zu Hause wurden von den Befragten dagegen nicht unterschiedlich bewertet.

**Table 2:** 

	Meinungsführer*innen		Nicht-Meinungsführer*innen		Signifikanz	Effektstärke
	M (SD)	*n*	M (SD)	*n*	*p*	Cohen’s d
Reste von Pflanzenschutzmitteln in Lebensmitteln, z. B. Glyphosat	4,4 (0,9)	124	3,8 (1,2)	729	0,000	0,566
Mikroplastik in Lebensmitteln	4,2 (1,2)	129	3,9 (1,2)	710	0,006	0,250
Schimmelpilzgifte in Lebensmitteln	4,0 (1,2)	133	3,5 (1,3)	622	0,000	0,400
Resistenzen gegen Antibiotika	4,0 (1,3)	126	3,9 (1,3)	757	0,701	0,077
Gentechnisch veränderte Lebensmittel	3,9 (1,2)	132	3,4 (1,4)	751	0,000	0,383
Bisphenol A in Lebensmittelverpackungen und -behältnissen	3,8 (1,2)	78	3,5 (1,3)	265	0,016	0,240
Mineralöle in Lebensmitteln	3,8 (1,4)	98	3,6 (1,3)	528	0,186	0,148
Lebensmittelhygiene in der Gastronomie	3,7 (1,2)	140	3,2 (1,2)	795	0,000	0,417
Aluminium in Lebensmittelverpackungen und -behältnissen	3,7 (1,2)	127	3,3 (1,3)	706	0,005	0,320
Bakterien in Lebensmitteln, z. B. Salmonellen, Campylobacter	3,5 (1,6)	138	3,4 (1,3)	790	0,747	0,069
Pyrrolizidinalkaloide in Tees oder Honig	3,4 (1,5)	56	3,5 (1,2)	172	0,559	−0,074
Nahrungsergänzungsmittel	2,5 (1,5)	138	2,5 (1,2)	765	0,812	0,000
Lebensmittelhygiene zu Hause	2,3 (1,4)	140	2,1 (1,2)	817	0,160	0,153

Die Signifikanzwerte wurden mittels des t‑Tests für Mittelwertunterschiede (M) berechnet

*1* = nicht beunruhigt, *5* = sehr beunruhigt

*n* Fallzahl; *M* Mittelwert; *SD* Standardabweichung

Ein deutlicher Unterschied zeigt sich zudem beim Interesse für Gesundheitsthemen im Lebensmittelbereich, welches bei Meinungsführer*innen signifikant höher liegt als bei anderen Personen (Tab. 3). So erreichen Meinungsführer*innen auf der 5‑stufigen Skala im Mittel einen Wert von M = 4,6 und liegen damit einen ganzen Skalenpunkt über dem durchschnittlichen Themeninteresse der Nicht-Meinungsführer*innen (M = 3,6). Gleichzeitig stufen sich Meinungsführer*innen jedoch nur als minimal weniger risikobereit ein als Nicht-Meinungsführer*innen, wobei dieser Unterschied aufgrund der geringen Effektstärke praktisch vernachlässigbar ist.

**Table 3:** 

	Meinungsführer*innen		Nicht-Meinungsführer*innen		Signifikanz	Effektstärke
	M (SD)	*n*	M (SD)	*n*	*p*	Cohen’s d
*Themeninteresse* (1 = interessiert mich gar nicht, 5 = interessiert mich sehr)	4,6 (0,7)	140	3,6 (1,0)	816	0,000	1,159
*Risikobereitschaft* (0 = gar nicht risikobereit, 10 = sehr risikobereit)	4,4 (2,6)	140	4,8 (2,1)	817	0,042	0,169

Die Signifikanzwerte wurden mittels des t‑Tests für Mittelwertunterschiede (M) berechnet

*n* Fallzahl; *M* Mittelwert; *SD* Standardabweichung

Auch bezüglich des Informationsverhaltens zu Gesundheitsthemen wurden Unterschiede zwischen Meinungsführer*innen und anderen Personen festgestellt. Meinungsführer*innen nutzen nahezu alle der abgefragten Informationskanäle signifikant häufiger als Nicht-Meinungsführer*innen (Abb. 2). Während fast alle Befragten über traditionelle Medien in den letzten 12 Monaten etwas über Gesundheitsthemen erfahren haben, werden spezialisierte Kanäle wie Fachzeitschriften von Meinungsführer*innen mehr als doppelt so häufig als Informationsquelle angegeben wie von anderen Personen.

Soziale Medien werden von 61 % der Meinungsführer*innen genutzt, während Nicht-Meinungsführer*innen nur zu 31 % auf dieses Medium zurückgreifen. Dies ist jedoch nicht der einzige Unterschied bei der Nutzung von sozialen Medien: Während unter den Meinungsführer*innen 24 % auch selbst Beiträge in sozialen Medien verfassen oder kommentieren, liegt dieser Anteil bei den Nicht-Meinungsführer*innen bei nur 12 %. Aufgrund des größeren Anteils der Nicht-Meinungsführer*innen in der Stichprobe nutzen jedoch, in absoluten Zahlen betrachtet, mehr Nicht-Meinungsführer*innen die sozialen Medien als Meinungsführer*innen.

Die bevorzugte Nutzung bestimmter Kanäle durch Meinungsführer*innen scheint hingegen nicht mit einem höheren Vertrauen in die Informationsquellen einherzugehen. Insbesondere Ärzt*innen, Behörden und der Politik stehen Meinungsführer*innen etwas kritischer gegenüber als Nicht-Meinungsführer*innen. Anders verhält es sich dagegen mit der Wissenschaft: Wie in Abb. 3 erkennbar, haben Meinungsführer*innen hier mehr Vertrauen als andere Personen, auch wenn beide Gruppen ein generell hohes Vertrauen in die Wissenschaft aufweisen. Das durchschnittlich geringste Vertrauen hatten alle Befragten dagegen in die Politik sowie in Lebensmittelhersteller.

## Fazit und Diskussion

Zwischen den themenspezifischen Meinungsführer*innen und Nicht-Meinungsführer*innen lassen sich Unterschiede in ihrer Risikowahrnehmung und ihrem Informationsverhalten bzgl. Gesundheitsthemen im Lebensmittelbereich erkennen. So haben Meinungsführer*innen auch von bislang vergleichsweise unbekannten Risiken häufiger gehört als andere. Zudem sind sie zumindest über einige Themen signifikant stärker beunruhigt als Nicht-Meinungsführer*innen. Sie greifen über die meisten Quellen häufiger auf Informationen zu Gesundheitsthemen zu, wobei vor allem eher spezialisierte Kanäle wie Fachzeitschriften oder Behörden verstärkt genutzt werden. Gleichzeitig sind Meinungsführer*innen bestimmten Institutionen wie Politik, Behörden oder Ärzt*innen gegenüber kritischer eingestellt als Nicht-Meinungsführer*innen, vertrauen dafür aber stärker der Wissenschaft.

Zwischen Meinungsführer*innen und anderen Personen gibt es zudem Unterschiede hinsichtlich ihrer Nutzung sozialer Medien. So sind Meinungsführer*innen nicht nur häufiger durch das Posten und Kommentieren von Beiträgen aktiv, sie nutzen soziale Medien auch häufiger als Nicht-Meinungsführer*innen als Informationsquelle für Gesundheitsthemen und können über diesen Kanal gut mit Risikoinformationen erreicht werden.

Wie die Ergebnisse gezeigt haben, sind Meinungsführer*innen für andere eine wichtige Informationsquelle. Hierbei muss jedoch beachtet werden, dass nach dem Social Amplification of Risk Framework die von den Meinungsführer*innen weitergegebenen Risikoinformationen die Risikowahrnehmung ihres Umfeldes verstärken oder abschwächen können – je nachdem, welche Haltung die Meinungsführer*innen selbst einnehmen. Da Meinungsführer*innen öffentlichen Institutionen tendenziell weniger stark vertrauen als Nicht-Meinungsführer*innen, kann dies auch bedeuten, dass Meinungsführer*innen die offiziellen Informationen stärker hinterfragen. Teilweise sind sie beunruhigter als andere über Risikothemen, die möglicherweise aus wissenschaftlicher Sicht kaum gesundheitsgefährdend sind. Bei anderen Themen unterschätzen sie hingegen die Risiken. Für öffentliche Einrichtungen stellt dies eine besondere Herausforderung dar, die bei der Entwicklung entsprechender Kommunikationsmaßnahmen bedacht werden muss. Eine Möglichkeit besteht darin, in der Kommunikation stärker Wissenschaftler*innen zu Wort kommen zu lassen, da die Wissenschaft bei den Meinungsführer*innen ein deutlich höheres Vertrauen genießt. Eine weitere Option ist, gerade in sozialen Medien die eigenen Inhalte für die jeweiligen Zielgruppen so verständlich und attraktiv aufzubereiten, dass die Nutzer*innen eher bereit sind, die Infografiken oder ähnlichen Inhalte direkt an ihr Umfeld weiterzuleiten, ohne die Inhalte vorher nochmal selbst zu überarbeiten. Hierdurch kann auch das Risiko aufgefangen werden, dass Risikobotschaften durch Missverständnisse seitens der Meinungsführer*innen so stark verändert werden, dass die eigentliche Botschaft nur noch verzerrt wiedergegeben wird.

Die vorliegende Studie hat auch gezeigt, dass der Zusammenhang zwischen der (themenbezogenen) Meinungsführerschaft und der Risikowahrnehmung im Lebensmittelbereich noch detaillierter untersucht werden sollte. So konnte in der Studie nicht näher analysiert werden, wodurch sich die Themen, bei denen Unterschiede in der Risikowahrnehmung von Meinungsführer*innen und Nicht-Meinungsführer*innen auftreten, voneinander unterscheiden. Dies könnte wertvolle Hinweise dafür liefern, bei welchen Themen Meinungsführer*innen im Lebensmittelbereich die eigene Kommunikation unterstützen können. Eine Analyse der Kommunikationsaktivitäten von Meinungsführer*innen zu den ausgewählten Themen kann zudem weiteren Aufschluss dazu liefern, wie konkret diese Beeinflussungsprozesse ablaufen.
